# Association between lactate-to-albumin ratio and in-hospital mortality in ICU patients with acute ischemic stroke: a retrospective cohort study

**DOI:** 10.3389/fneur.2026.1908031

**Published:** 2026-09-17

**Authors:** Xiaocui Yang, Yue Zhang, Min Ou, Yiwen Liu

**Affiliations:** West China Hospital, Sichuan University, Chengdu, Sichuan, China

**Keywords:** in-hospital mortality, ischemic stroke, lactate-to-albumin ratio, logistic regression, retrospective cohort study

## Abstract

**Background:**

Early risk stratification is critical for critically ill patients with ischemic stroke. The prognostic value of the lactate-to-albumin ratio (LAR) remains unclear in this population.

**Methods:**

This single-center retrospective cohort study enrolled adult ICU patients with ischemic stroke from West China Hospital. LAR was calculated using the first lactate and albumin values on ICU admission. Log-transformed LAR was adopted as the main exposure, and the primary outcome was in-hospital mortality. Multivariable logistic regression, ROC analysis, quartile stratification, subgroup, and sensitivity analyses were performed among 749 patients with complete exposure and outcome data, of whom 576 had complete covariate data and comprised the primary multivariable regression cohort.

**Results:**

A total of 749 patients were included, with an in-hospital mortality rate of 30.7%. In complete-case multivariable analysis (*N* = 576; deaths = 172), log-transformed LAR was independently associated with in-hospital mortality (OR = 1.44, 95%CI: 1.09–1.91, *p* = 0.010) after full adjustment. Each two-fold increase in LAR was associated with a 29% increase in mortality odds. The highest LAR quartile had a significantly higher mortality risk than the lowest quartile (OR = 2.16, 95%CI: 1.21–3.92). LAR showed modest predictive ability (AUC = 0.628). When modeled as a raw continuous LAR, the direction of association remained consistent but did not reach conventional statistical significance (OR 1.23; 95% CI 0.97–1.56; *p* = 0.083). Moreover, no significant interaction or non-linearity was found.

**Conclusion:**

Elevated LAR is independently linked to higher in-hospital mortality among critically ill ischemic stroke patients. It may serve as a simple adjunctive marker for early risk stratification.

## Introduction

Ischemic stroke remains one of the leading causes of death and long-term disability worldwide. Although many patients with ischemic stroke can be managed in stroke units or general wards, a clinically important subset requires ICU-level care (1, 2). Mortality risk in this population is driven not only by the primary cerebral ischemic injury but also by systemic physiological derangements that accompany critical illness. Consequently, simple and reproducible biomarkers obtained early after ICU admission may help clinicians identify patients at high risk and contextualize prognosis alongside established clinical severity scores.

Serum lactate is widely used in critical care as a marker of tissue hypoperfusion, cellular oxygen imbalance, mitochondrial dysfunction, adrenergic stress, and global metabolic disturbance. In stroke, lactate may also reflect the metabolic consequences of ischemic brain injury and systemic stress responses. Serum albumin is another routinely available laboratory measurement with potential prognostic relevance. Albumin reflects nutritional state, systemic inflammation, capillary permeability, hepatic synthesis, renal or gastrointestinal loss, and chronic disease burden. In patients with acute ischemic stroke, hypoalbuminemia has been associated with worse outcomes in several studies (3). Albumin also has biological properties that may be relevant to acute brain injury, including antioxidant and anti-inflammatory effects, maintenance of oncotic pressure, and possible modulation of endothelial function (4). The lactate-to-albumin ratio (LAR) combines these two biomarkers into a single index. Conceptually, a high LAR may identify patients with simultaneous metabolic stress and reduced physiological reserve. LAR has been investigated in sepsis, acute kidney injury, intracerebral hemorrhage, traumatic brain injury, myocardial infarction, and other critically ill populations (5–8). Recent studies using large critical care databases have also examined the association between LAR and short-term mortality in patients with ischemic stroke (9–11). These studies generally support the prognostic relevance of LAR, but important questions remain regarding cohort definition, exposure modeling, discrimination, non-linear patterns, and the stability of the association across clinically relevant subgroups.

This single-center retrospective cohort study was conducted using clinical data from West China Hospital. We aimed to explore the association between LAR and in-hospital mortality among adult patients with ischemic stroke admitted to the ICU. We hypothesized that higher log-transformed LAR would be independently associated with in-hospital mortality. We further aimed to evaluate the discriminatory performance of LAR, examine the risk associated with LAR quartiles, assess subgroup consistency, and perform sensitivity analyses to determine whether conclusions were robust to alternative exposure definitions.

## Methods

### Study design and data source

This was a single-center retrospective cohort study. All clinical data were extracted from the electronic medical record system of West China Hospital from March 2023 to October 2025, including demographic characteristics, vital signs, laboratory test results, disease severity scores, diagnostic information, treatment regimens, and hospital outcomes. Since only de-identified clinical data were used in this study, individual informed consent was waived. The reporting of this study complied with the Strengthening the Reporting of Observational Studies in Epidemiology (STROBE) guidelines for observational research. The reporting structure of the article was developed with reference to the STROBE guidance for observational studies (12).

The study enrolled adult patients aged 18 years or older, with an ICU length of stay of no less than 1 day and a confirmed diagnosis of ischemic stroke. Participants were screened according to highly specific diagnostic codes for ischemic stroke (ICD-10-CM; full list in Supplementary Table S3). Patients with mixed or primarily hemorrhagic cerebrovascular diagnoses, transient ischemic attack diagnoses, subarachnoid hemorrhage, epidural or subdural hematoma, and other non-ischemic or ambiguous stroke-related diagnostic categories were excluded. The disease codes correspond to ischemic stroke and selected ischemic stroke subtypes or etiologies, including atherosclerotic, cardioembolic, and cervicocerebral artery dissection-related ischemic stroke categories. The exported analysis file did not contain patient-level diagnostic code fields.

The initial diagnosis-based extraction identified 5,414 eligible adult ICU patients with ischemic stroke diagnoses. Because the research question required calculation of LAR, patients needed both a first lactate measurement and a first albumin measurement after ICU admission. A total of 759 patients had both measurements available. Ten patients lacked hospital discharge status and were excluded from outcome analyses. The final primary analytical cohort therefore included 749 patients with first lactate and first albumin measurements and in-hospital outcome information. The complete-case multivariable regression cohort included 576 patients with non-missing values for log(LAR), age, sex, APACHE IV score, Glasgow Coma Scale, SOFA score, and hospital mortality.

### Exposure definition and laboratory measurements

The primary exposure was LAR, calculated as first serum lactate divided by first serum albumin after ICU admission. All laboratory indicators used in this study, including lactate, albumin, electrolytes, blood cell count, blood urea nitrogen, creatinine, hemoglobin, and platelet count, represented the first available laboratory measurements obtained within 24 h after ICU admission. Laboratory values obtained in the emergency department or general ward before ICU admission were excluded. The first available measurement within this window was used for each analyte. Exact timestamps for individual lactate and albumin measurements were not retained in the analysis. Lactate was measured in mmol/L and albumin in g/dL. LAR was calculated dimensionally using the values recorded. Because LAR is a ratio derived from two clinically meaningful markers, it was examined both as a continuous variable and as a quartile. In the ICU setting, lactate is typically ordered when clinicians suspect hemodynamic instability, sepsis, shock, tissue hypoperfusion, or severe metabolic derangement; it is not part of routine stroke-specific laboratory panels. Albumin is often included in comprehensive metabolic or liver function panels ordered for patients with suspected systemic illness, nutritional compromise, or fluid management needs. In an ischemic-stroke ICU population, many patients with stable, uncomplicated strokes do not trigger these ordering indications, which may explain why only 14% of diagnosis-eligible patients had both measurements available.

Exploratory distributional assessment showed that LAR was markedly right-skewed. In the 749-patient analytic cohort, LAR had a median of 0.56, an interquartile range of approximately 0.36–0.90, a mean of 0.85, and a maximum of 11.73. The right-skewed distribution was driven by a small number of patients with high lactate and/or low albumin values. Because a raw continuous LAR variable may be sensitive to extreme right-tail observations, natural log-transformed LAR was used as the primary exposure in multivariable regression analyses. This approach was selected because it better reflects the empirical distribution of the biomarker and produces an effect estimate that is interpretable as a multiplicative change in LAR (13). For clinical interpretability, we also calculated the odds ratio for each 2-fold increase in LAR. LAR quartiles were used to describe baseline characteristics and to evaluate risk gradients across exposure categories.

### Outcome definition

The primary outcome was in-hospital mortality. In-hospital mortality was defined using hospital discharge status. Patients with missing hospital discharge status were excluded from outcome analyses. In the final analytic cohort of 749 patients, 519 survived hospital discharge, and 230 died in hospital.

### Covariates

Covariates were selected based on clinical relevance, prior literature, data availability, and the need to avoid excessive model complexity. The primary fully adjusted model included age, sex, APACHE IV score, Glasgow Coma Scale (GCS), and Sequential Organ Failure Assessment (SOFA) score. Age and sex were included as basic demographic confounders. GCS was included because neurological status is central to prognosis in ischemic stroke. SOFA score was included to adjust for non-neurological organ dysfunction. The APACHE IV score was included as a global critical illness severity measure. Although APACHE IV, GCS, and SOFA may contain partially overlapping prognostic information, retaining them allowed simultaneous adjustment for overall physiologic severity, neurological impairment, and organ dysfunction.

The missingness of APACHE IV, GCS, and SOFA scores was attributable to clinical circumstances—sedation or mechanical ventilation precluding GCS assessment, incomplete admission workups in transferred or rapidly discharged/deceased patients limiting APACHE IV, and absent organ-system component assessments limiting SOFA—consistent with severity-related rather than random missingness.

Additional variables were used for descriptive or subgroup analyses. Acute kidney injury (AKI) was defined as any AKI diagnosis recorded during the ICU stay, not restricted to admission status. Mechanical ventilation, diabetes mellitus, and hypertension were coded as binary variables indicating their presence at any time during admission. In the exported data, these indicators appeared as 1 when present and missing otherwise. Because the variables contained only 1 and missing values, missing values were interpreted as the absence of the corresponding documented condition or support status and recoded as 0. These variables were extracted from structured database fields using a presence-only coding structure, in which conditions are recorded as 1 when documented and otherwise as missing (no explicit 0 values are present in the raw database). To evaluate the potential impact of this coding, we performed sensitivity analyses in which missing values were alternatively treated as a separate category. This approach was consistent with the structure of the extracted variables but remains a potential source of misclassification. Additional first laboratory and clinical variables were summarized in supplementary descriptive analyses, including heart rate, respiratory rate, sodium, potassium, blood urea nitrogen, creatinine, white blood cell count, hemoglobin, platelet count, ICU length of stay, and hospital length of stay.

A directed acyclic graph (DAG) illustrating the causal framework for covariate selection is provided in Supplementary Figure S2.

### Statistical analysis

Continuous variables were summarized as medians and interquartile ranges because many clinical and laboratory variables were not normally distributed. Categorical variables were summarized as counts and percentages. Baseline characteristics were compared across LAR quartiles using the Kruskal–Wallis rank-sum test for continuous variables and Pearson’s chi-squared test for categorical variables. Missing values were not imputed; variable-specific summaries were used for available observations. Complete-case analysis was used for multivariable logistic regression.

The primary association between log-transformed LAR and in-hospital mortality was evaluated using logistic regression. Three models were constructed. Model 1 was an unadjusted model containing only log(LAR). Model 2 was adjusted for age and sex. Model 3, the primary fully adjusted model, adjusted for age, sex, APACHE IV score, GCS, and SOFA score. Results were reported as odds ratios (ORs) and 95% confidence intervals (CIs). A clinically interpretable effect estimate was calculated for each two-fold increase in LAR using the coefficient for log(LAR). Because a one-unit increase in natural log-transformed LAR corresponds to an approximately 2.718-fold increase in LAR, the doubling estimate was calculated by exponentiating the log(LAR) coefficient multiplied by log(2).

Collinearity among severity scores was assessed using Spearman correlation coefficients and variance inflation factors (VIFs). To evaluate the robustness of the LAR association to the choice of severity adjustment, sensitivity analyses were performed with all possible combinations of APACHE IV, GCS, and SOFA scores. Covariate selection was guided by a confounder-adjustment framework, including variables associated with both LAR and in-hospital mortality that were not on the causal pathway from LAR to mortality.

Receiver operating characteristic (ROC) curve analysis was performed to evaluate the discriminatory ability of LAR for in-hospital mortality. The area under the ROC curve (AUC) and 95% CI were calculated using DeLong’s method. Because log transformation preserves the ordering of LAR values, ROC discrimination for raw LAR and log(LAR) is equivalent for practical purposes. The ROC result was interpreted cautiously; AUC values approximately 0.6–0.7 indicate limited or modest discrimination rather than strong predictive performance (14). Therefore, LAR was considered an adjunctive risk marker rather than a stand-alone prediction model.

To visualize risk across clinically interpretable exposure categories, patients were divided into LAR quartiles. A multivariable logistic model compared Q2, Q3, and Q4 with Q1 as the reference category, adjusting for age, sex, APACHE IV score, GCS, and SOFA score. Adjusted ORs and 95% CIs were plotted in a black-and-white forest-style figure. Restricted cubic spline analysis with four knots placed at the 5th, 35th, 65th, and 95th percentiles was performed for log(LAR) to evaluate whether the association with in-hospital mortality was non-linear. The overall association and non-linear component were assessed using Wald statistics. Because the non-linear component was not statistically significant, the RCS result was summarized as a sensitivity analysis.

Prespecified subgroup analyses were performed by age (<65 vs. ≥65 years), sex, AKI status, and mechanical ventilation status. Interaction terms between log(LAR) and subgroup variables were tested using likelihood ratio tests comparing models with and without the interaction term. Subgroup analyses were considered exploratory and were interpreted primarily through interaction *p*-values rather than by the statistical significance of individual subgroup estimates.

Sensitivity analyses included the primary log(LAR) model, the LAR doubling estimate, the raw continuous LAR model, the comparison of Q4 vs. Q1, and RCS tests for overall association and non-linearity. Complete-case analysis was used for the primary multivariable logistic regression. To evaluate whether missing severity-score data could bias the primary estimate, we quantified the missingness patterns of APACHE IV, GCS, and SOFA scores, compared baseline characteristics between patients included (*n* = 576) and excluded (*n* = 173) from the complete-case analysis, and performed a multiple imputation by chained equation sensitivity analysis. Twenty imputed datasets were generated with posterior predictive draws; these imputation models included the outcome, the exposure, and all analysis covariates. Logistic regression estimates across the imputed datasets were pooled using Rubin’s rules.

All tests were two-sided, and *p*-values less than 0.05 were considered statistically significant. Statistical analyses were performed using R software 4.3.1 (R Foundation for Statistical Computing, Vienna, Austria). Key packages included “gtsummary” and “flextable” for table generation, “pROC” for ROC analysis and DeLong’s test, “rms” for restricted cubic spline modeling, “mice” for multiple imputation, “forestplot” for forest plots, and “ggplot2” for data visualization.

## Results

### Cohort selection and outcome frequency

The diagnosis-based extraction identified 5,414 adult ICU patients with ischemic stroke diagnoses.

After applying prespecified inclusion and exclusion criteria, 749 patients with complete LAR and outcome data formed the primary analytical cohort, of whom 576 had full covariate data and were included in the fully adjusted multivariable analysis. A detailed patient flow diagram is provided in Supplementary Figure S1. Complete-case modeling was retained because these variables were central to confounding adjustment and because the number of outcome events remained sufficient for the prespecified model. We compared baseline characteristics and outcomes between all 5,414 diagnosis-eligible ICU patients and the 759 patients who had both lactate and albumin measurements available. Patients included in the analytic cohort were substantially more severely ill than those excluded (Supplementary Table S5).

Among 749 patients, 92 patients (12.3%) were missing all three severity scores, 43 (5.7%) were missing GCS only, and 33 (4.4%) were missing the APACHE IV score only. The remaining covariates had the following missingness: heart rate 4.8%, respiratory rate 5.5%, temperature 2.7%, SpO2 20.2%, BUN 0.3%, hemoglobin 0.3%, platelets 0.3%, WBC 0.3%, AKI 26.2%, mechanical ventilation 38.9%, diabetes mellitus 91.5%, hypertension 65.8%, and hospital admission source 27.6%. Lactate and albumin had no missing values by definition of cohort inclusion. A complete missingness table has been added to Supplementary Table S6.

### Baseline characteristics according to LAR quartiles

Patients were divided into four LAR quartiles. Quartile sizes were balanced, with 188 patients in Q1 and 187 patients in each of Q2, Q3, and Q4. LAR increased progressively across quartiles: median LAR was 0.27 in Q1, 0.44 in Q2, 0.70 in Q3, and 1.57 in Q4. Median lactate increased from 0.8 mmol/L in Q1 to 3.9 mmol/L in Q4, whereas median albumin decreased from 3.3 g/dL in Q1 to 2.5 g/dL in Q4 (Table 1).

**Table 1:** Baseline characteristics according to LAR quartiles.

Characteristic	Q1	Q2	Q3	Q4	*p* value
	*N* = 188	*N* = 187	*N* = 187	*N* = 187	
Age, years	64.0 (53.0–76.5)	68.0 (57.0–77.0)	65.0 (56.0–75.0)	66.0 (56.0–76.0)	0.290
Sex					0.466
Female	81 (43%)	78 (42%)	71 (38%)	86 (46%)	
Male	107 (57%)	109 (58%)	116 (62%)	101 (54%)	
Lactate, mmol/L	0.8 (0.7–1.0)	1.3 (1.1–1.5)	2.0 (1.7–2.4)	3.9 (2.9–5.4)	<0.001
Albumin, g/dL	3.3 (2.9–3.6)	3.0 (2.6–3.4)	2.8 (2.4–3.4)	2.5 (2.1–3.0)	<0.001
LAR	0.27 (0.23–0.32)	0.44 (0.40–0.50)	0.70 (0.63–0.80)	1.57 (1.13–2.25)	<0.001
APACHE IV score	59.0 (45.0–81.0)	66.0 (49.0–84.0)	72.5 (54.0–95.0)	92.0 (71.0–112.0)	<0.001
Glasgow Coma Scale	14.0 (10.0–15.0)	13.0 (9.0–15.0)	13.0 (9.0–15.0)	10.0 (7.0–14.0)	<0.001
SOFA score	5.0 (3.0–7.0)	5.0 (3.0–7.0)	6.0 (4.0–8.0)	7.0 (5.0–10.0)	<0.001
Acute kidney injury, n (%)	140 (74%)	132 (71%)	129 (69%)	152 (81%)	0.033
Mechanical ventilation, n (%)	98 (52%)	114 (61%)	110 (59%)	136 (73%)	<0.001
In-hospital mortality					<0.001
Survivor	148 (79%)	142 (76%)	127 (68%)	102 (55%)	
Non-survivor	40 (21%)	45 (24%)	60 (32%)	85 (45%)	

LAR, lactate-to-albumin ratio; APACHE IV, Acute Physiology and Chronic Health Evaluation IV; GCS, Glasgow Coma Scale; SOFA, Sequential Organ Failure Assessment.

Continuous variables are presented as median (interquartile range); categorical variables are presented as *n* (%).

*p*-values were calculated using the Kruskal–Wallis rank-sum test for continuous variables and Pearson’s chi-squared test for categorical variables.

Median APACHE IV score increased from 59.0 in Q1 to 92.0 in Q4. Median SOFA score increased from 5.0 to 7.0, and median GCS decreased from 14.0 to 10.0. AKI was common in all quartiles and was most frequent in Q4 (81%). Mechanical ventilation also increased across LAR quartiles, from 52% in Q1 to 73% in Q4.

In-hospital mortality increased monotonically across the LAR quartiles. Mortality was 21% in Q1, 24% in Q2, 32% in Q3, and 45% in Q4. The difference across the quartiles was statistically significant. In Supplementary Table S1, high-LAR patients had higher heart rate, blood urea nitrogen, creatinine, and white blood cell count.

### Association between log(LAR) and in-hospital mortality

In logistic regression, log-transformed LAR was consistently associated with in-hospital mortality across progressively adjusted models (Table 2). In the unadjusted model, log(LAR) was strongly associated with mortality (OR 1.95; 95% CI 1.53–2.50; *p* < 0.001). After adjustment for age and sex, the association remained essentially unchanged (OR 1.97; 95% CI 1.54–2.52; *p* < 0.001). In the fully adjusted model, which included age, sex, APACHE IV score, GCS, and SOFA score, the magnitude of association was attenuated but remained statistically significant (OR 1.44; 95% CI 1.09–1.91; *p* = 0.010). For clinical interpretation, the coefficient for log(LAR) was converted into an effect estimate per two-fold increase in LAR. Each two-fold increase in LAR was associated with 29% higher odds of in-hospital mortality after full adjustment (OR 1.29; 95% CI 1.06–1.56; *p* = 0.010). We performed an IPW analysis to address selection bias related to the availability of exposure measurements. A propensity score for inclusion in the 759-patient analytic cohort was estimated using logistic regression with age, sex, AKI status, mechanical ventilation, APACHE IV score, GCS, and SOFA score as predictors. After accounting for selection bias via IPW, the association between log(LAR) and in-hospital mortality remained statistically significant [OR 1.74 (95% CI 1.20–2.54; *p* = 0.004)].

**Table 2:** Association between log-transformed LAR and in-hospital mortality.

Model	Adjusted covariates	OR (95% CI)	*p* value
Model 1	None	1.95 (1.53–2.50)	<0.001
Model 2	Age, sex	1.97 (1.54–2.52)	<0.001
Model 3	Age, sex, APACHE IV score, GCS, SOFA	1.44 (1.09–1.91)	0.010

LAR, lactate-to-albumin ratio; OR, odds ratio; CI, confidence interval; APACHE IV, Acute Physiology and Chronic Health Evaluation IV; GCS, Glasgow Coma Scale; SOFA, Sequential Organ Failure Assessment.

Model 1 was unadjusted; model 2 was adjusted for age and sex; model 3 was adjusted for age, sex, APACHE IV score, Glasgow Coma Scale, and SOFA score. Model 3 was considered the primary fully adjusted model.

### LAR quartiles and adjusted mortality risk

To examine the relationship between clinically interpretable LAR categories and mortality risk, LAR quartiles were modeled with Q1 as the reference group. After adjustment for age, sex, APACHE IV score, GCS, and SOFA score, there was a significant linear trend of increasing mortality risk across LAR quartiles (p-for-trend = 0.007). Q2 and Q3 had higher point estimates than Q1, but none reached statistical significance. Q2 had an OR of 1.28 (95% CI 0.71–2.34), and Q3 had an OR of 1.53 (95% CI 0.86–2.77). In contrast, Q4 was significantly associated with increased in-hospital mortality (OR 2.16; 95% CI 1.21–3.92; *p* = 0.010) (Figure 1). The quartile analysis is also consistent with the descriptive mortality gradient observed in Table 1.

**Figure 1:**
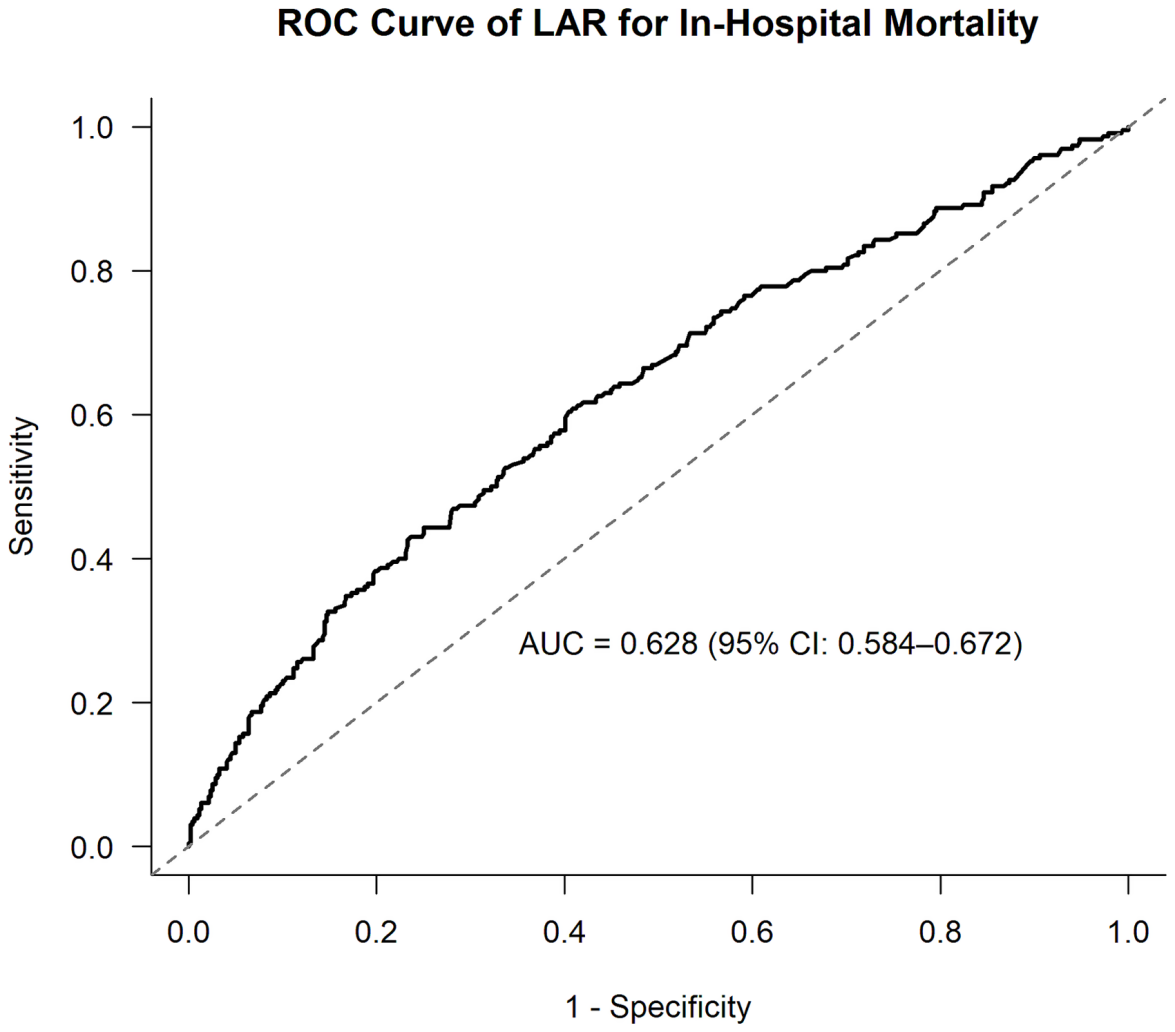
Adjusted odds ratios for in-hospital mortality according to LAR quartiles. Odds ratios were adjusted for age, sex, APACHE IV score, Glasgow Coma Scale, and SOFA score. Q1 was used as the reference group.

### Discriminatory performance of LAR

ROC analysis showed that LAR had an AUC of 0.628 for in-hospital mortality, with a 95% CI of 0.584 to 0.672 (Figure 2).

**Figure 2:**
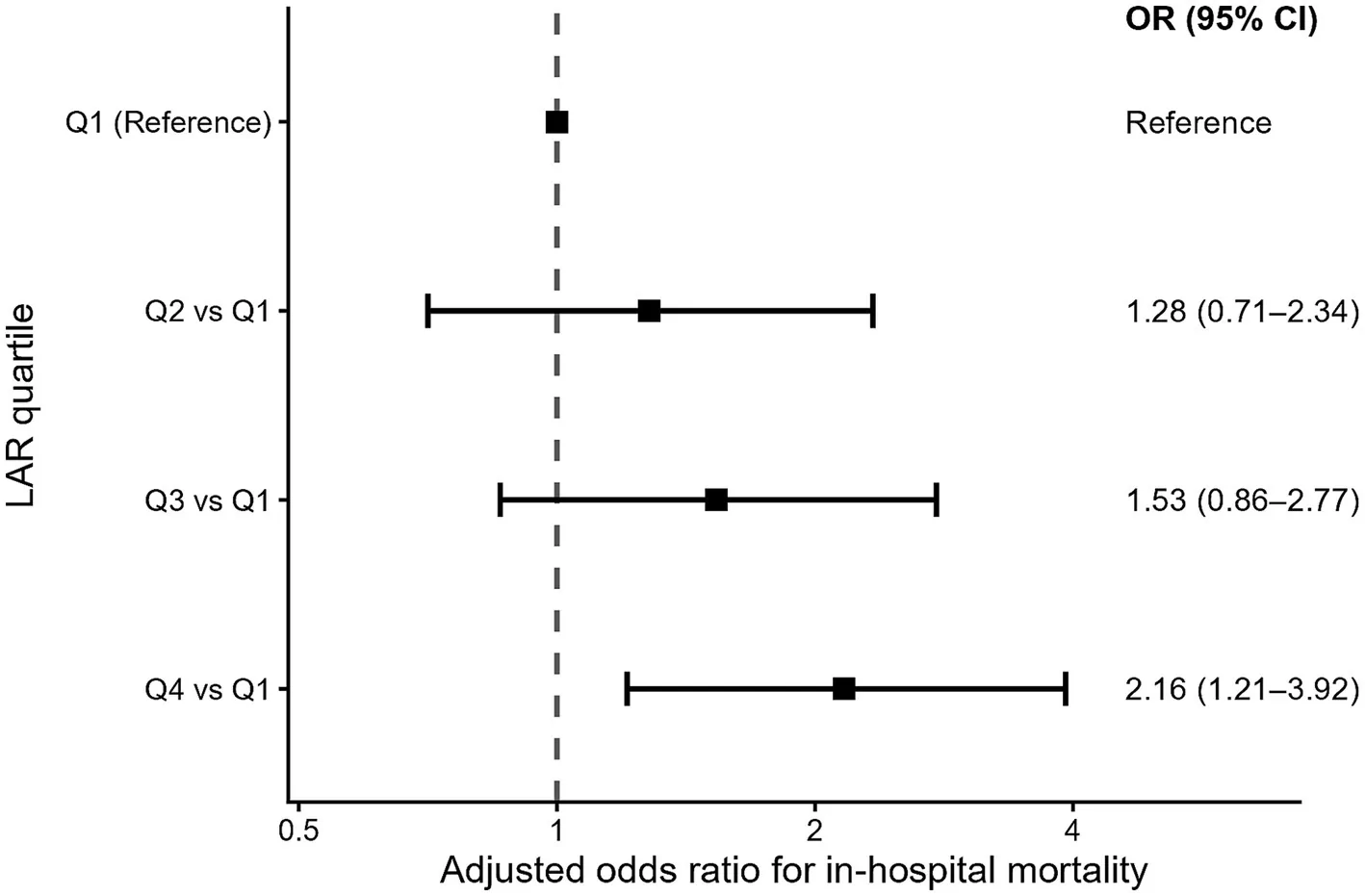
ROC curve of LAR for predicting in-hospital mortality. LAR showed modest discriminatory ability, with an AUC of 0.628 (95% CI 0.584–0.672).

Using the Youden index, the optimal LAR cutoff was 0.60, yielding a sensitivity of 62.8% and specificity of 61.1% for in-hospital mortality (PPV 39.5%, NPV 81.0%). At higher cutoffs, specificity increased to 84.7% but sensitivity decreased to 34.3%.

Spearman correlations indicated moderate inter-correlations among the three severity measures (APACHE IV vs. SOFA: rho = 0.612; APACHE IV vs. GCS: rho = −0.492; SOFA vs. GCS: rho = −0.485), and weak-to-moderate correlations between log(LAR) and each score (vs. APACHE IV: rho = 0.352; vs. SOFA: rho = 0.302; vs. GCS: rho = −0.173). In the fully adjusted model, VIFs were low for all predictors (APACHE IV: 2.23, SOFA: 1.96, GCS: 1.46, log LAR: 1.21, age: 1.15, sex: 1.03), indicating no meaningful multicollinearity.

In an incremental discrimination analysis, adding log(LAR) to a base model containing age, sex, APACHE IV score, GCS, and SOFA score increased the AUC from 0.729 (95% CI 0.681–0.776) to 0.739 (95% CI 0.692–0.787), corresponding to a small, statistically non-significant improvement (ΔAUC + 0.011; 95% CI −0.003 to 0.025; *p* = 0.061). These findings indicate that LAR captures limited incremental prognostic information beyond established severity scores and should be regarded as an adjunctive rather than a stand-alone risk marker.

In single-variable analysis, LAR demonstrated modest discrimination (AUC 0.642; 95% CI 0.591–0.695), which was not significantly different from lactate alone (AUC 0.652) or established severity scores (APACHE IV 0.676, SOFA 0.678, GCS 0.685), but was superior to albumin alone (AUC 0.493). Adding LAR to the base model increased AUC from 0.729 to 0.739 (ΔAUC = +0.011; 95% CI −0.003 to 0.025), but this improvement was not statistically significant. The fully adjusted model demonstrated good calibration (Hosmer–Lemeshow *p* = 0.686).

In head-to-head comparison, log(lactate) was significantly associated with mortality after full adjustment (OR 1.59; 95% CI 1.18–2.14; *p* = 0.002), whereas log(albumin) was not (OR 1.48; 95% CI 0.65–3.40; *p* = 0.354). LAR (OR 1.44; *p* = 0.010) did not outperform lactate alone. A likelihood ratio test showed no significant difference between the model with both components and the model with LAR (*p* = 0.055).

### Subgroup analyses

Subgroup analyses examined whether the association between log (LAR) and in-hospital mortality varied by age, sex, AKI status, or mechanical ventilation status (Figure 3). In the complete-case subgroup dataset, there were 273 patients aged <65 years and 303 aged ≥65 years; 243 were female, and 333 were male; 140 had no AKI and 436 had AKI; 215 were not mechanically ventilated, and 361 were mechanically ventilated.

**Figure 3:**
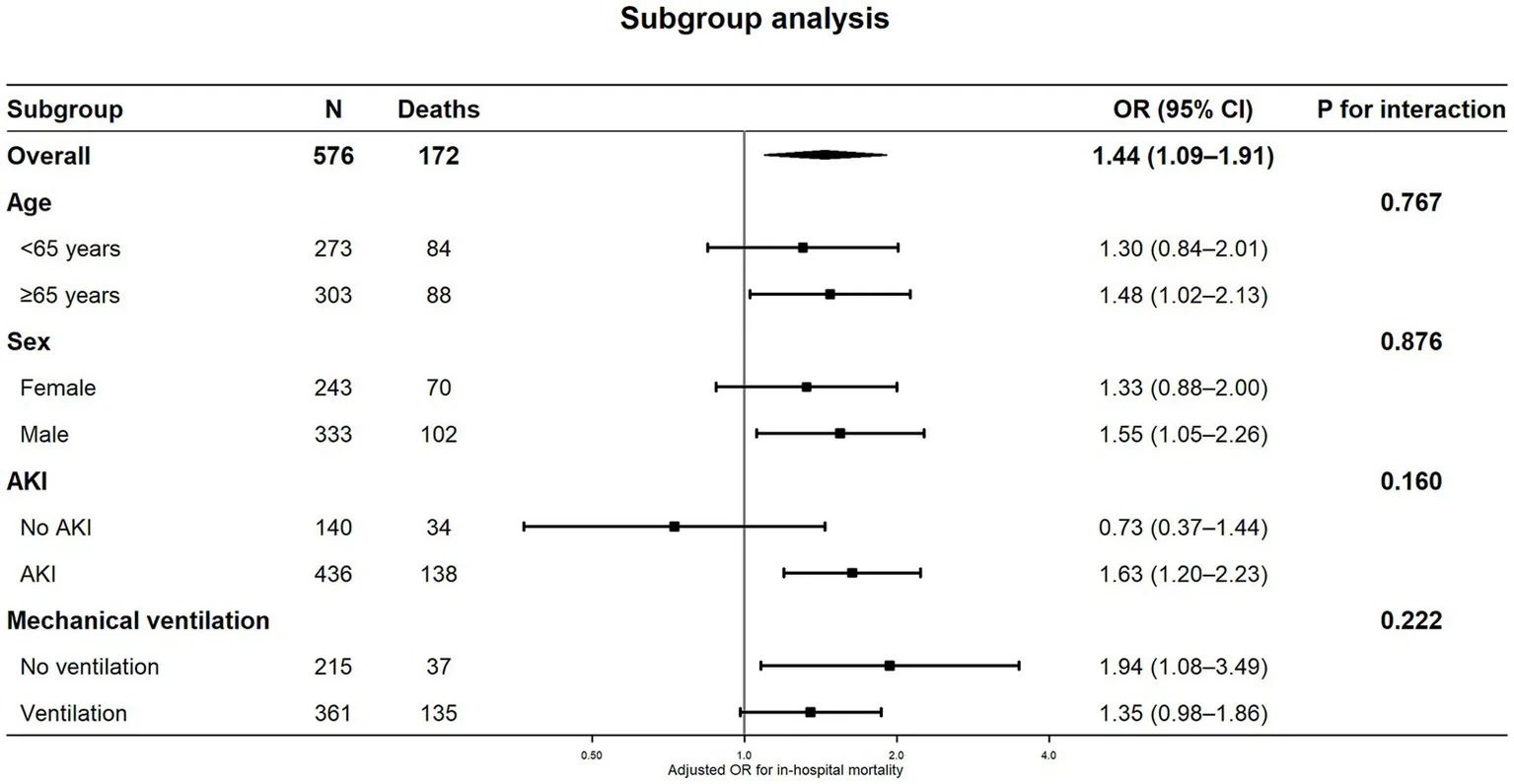
Subgroup analysis of the association between log-transformed LAR and in-hospital mortality. Odds ratios were adjusted for age, sex, APACHE IV score, Glasgow Coma Scale, and SOFA score, except when the stratification variable itself was used for subgrouping. No statistically significant interactions were observed across age, sex, AKI, or mechanical ventilation subgroups.

The estimated association between log (LAR) and mortality was directionally positive in most subgroups. Among patients aged ≥65 years, the OR was 1.48 (95% CI 1.02–2.13), whereas among those aged <65 years, the OR was 1.30 (95% CI 0.84–2.01). Among male patients, the OR was 1.55 (95% CI 1.05–2.26), whereas among female patients, the OR was 1.33 (95% CI 0.88–2.00). Among patients with AKI, the OR was 1.63 (95% CI 1.20–2.23); among those without AKI, the OR was 0.73 (95% CI 0.37–1.44). Among patients without mechanical ventilation, the OR was 1.94 (95% CI 1.08–3.49), whereas among mechanically ventilated patients, the OR was 1.35 (95% CI 0.98–1.86). Overall, subgroup analyses showed no statistically significant evidence of effect modification by age (*p* for interaction = 0.767), sex (*p* = 0.876), AKI status (*p* = 0.160), or mechanical ventilation status (*p* = 0.222).

### Missing data analysis

Among the 749 patients in the analytic cohort, 173 (23.1%) had at least one missing severity-score covariate and were excluded from the complete-case analysis. Missingness was driven by GCS (140 patients, 18.7%), APACHE IV score (130, 17.4%), and SOFA score (92, 12.3%); 92 patients (12.3%) were missing all three scores, while 43 (5.7%) were missing GCS only and 33 (4.4%) were missing APACHE IV score only. Patients excluded from complete-case analysis were comparable to included patients with respect to age, sex, lactate, albumin, LAR, mechanical ventilation, and in-hospital mortality (29.9% vs. 33.5%; *p* = 0.41), but had a lower prevalence of AKI (67.6% vs. 75.7%; *p* = 0.044) and were less frequently admitted to neurological ICUs. In a multiple imputation sensitivity analysis, the fully adjusted association of log(LAR) with in-hospital mortality remained significant (OR 1.54; 95% CI 1.20–1.97; *p* < 0.001; FMI = 12.0%), and the association for raw continuous LAR also reached significance (OR 1.35; 95% CI 1.11–1.64; *p* = 0.003), indicating that the primary conclusions were robust to the handling of missing data.

### Sensitivity analyses and non-linear assessment

Sensitivity analyses are summarized in Supplementary Table S2. The primary log (LAR) analysis showed a significant association with in-hospital mortality (OR 1.44; 95% CI 1.09–1.91; *p* = 0.010). The LAR doubling estimate was also significant (OR 1.29; 95% CI 1.06–1.56; *p* = 0.010), indicating a clinically interpretable effect. The raw continuous LAR model showed a directionally consistent association but did not reach conventional statistical significance (OR 1.23; 95% CI 0.97–1.56; *p* = 0.083). This finding supports the decision to use log(LAR) as the primary exposure because the raw LAR distribution was markedly right-skewed and potentially influenced by extreme values. A distributional figure comparing raw and log-transformed LAR has been added as Supplementary Figure S3. The quartile analysis also supports the main conclusion, with Q4 vs. Q1 showing a significant association with mortality (OR 2.16; 95% CI 1.21–3.92; *p* = 0.010).

Restricted cubic spline analysis showed an overall association between log (LAR) and in-hospital mortality (*p* = 0.041) but did not provide evidence of significant non-linearity (*p* for non-linearity = 0.463). Accordingly, no threshold or inflection point was claimed. Sensitivity analyses across all eight severity-score combinations yielded consistently significant associations between log(LAR) and mortality (Supplementary Table S7). Sensitivity analyses for binary variable coding.

The primary association between log(LAR) and in-hospital mortality was consistent across alternative treatments of missing binary variables (AKI and mechanical ventilation): OR 1.44 (95% CI 1.09–1.91) when missing values was coded as absence, OR 1.44 (1.09–1.91) when missing values was treated as a separate category, OR 1.53 (1.07–2.19) in complete-case analysis (*n* = 295), and OR 1.51 (range 1.49–1.54) after multiple imputation including these variables.

## Discussion

In this retrospective cohort of adult ICU patients with ischemic stroke who had first lactate and albumin measurements available, higher LAR was associated with increased in-hospital mortality. Several lines of evidence support this conclusion. First, in-hospital mortality increased across LAR quartiles, from 21% in the lowest quartile to 45% in the highest quartile. Second, log-transformed LAR remained significantly associated with in-hospital mortality after adjustment for age, sex, APACHE IV score, GCS, and SOFA score. Third, the association was clinically interpretable: each two-fold increase in LAR corresponded to approximately 29% higher odds of in-hospital mortality. Fourth, the highest LAR quartile had more than twice the adjusted odds of in-hospital mortality compared with the lowest quartile. Finally, subgroup analyses showed no statistically significant interactions by age, sex, AKI, or mechanical ventilation status. However, our findings should be interpreted as applying to the subgroup of critically ill ischemic-stroke patients in whom lactate and albumin were clinically indicated, rather than to all ICU patients with ischemic stroke. Generalization to unselected stroke ICU populations should be made with caution.

At the same time, the AUC of LAR for in-hospital mortality was 0.628, indicating modest discrimination. LAR is therefore not a stand-alone mortality prediction tool. It should be understood as an accessible biomarker that contributes prognostic information and may help identify a high-risk physiological phenotype. The RCS analysis did not show significant non-linearity, so we did not claim a threshold effect. The raw LAR model was directionally consistent but not statistically significant after full adjustment, reinforcing the importance of accounting for right-skewed biomarker distributions.

Our findings are broadly consistent with prior studies examining LAR in ischemic stroke and other critically ill populations. A MIMIC-IV study of ischemic stroke patients without reperfusion therapy reported that LAR was independently associated with 28-day all-cause mortality and used multivariable modeling, ROC analysis, restricted cubic splines, and subgroup analyses (9). Another study reported that LAR predicted 28-day mortality in acute ischemic stroke patients and suggested that the ratio may perform better than lactate or albumin alone (10). A more recent MIMIC-IV analysis of critically ill patients with ischemic stroke also examined short- and long-term mortality in relation to LAR (11). The prognostic performance of LAR is known to vary across clinical contexts. For example, in a recent coronary care unit cohort of patients with acute decompensated heart failure, LAR was associated with mortality in unadjusted analysis but not after multivariable adjustment, illustrating that the independent prognostic contribution of LAR depends on population characteristics and confounding adjustment (15). These studies provide an important context for our analysis and serve as a structural template for organizing the present study. The present study differs in several ways. First, the cohort definition emphasized high-specificity ischemic stroke-related diagnosis codes and excluded mixed, hemorrhagic, TIA, and ambiguous diagnoses. Second, the primary exposure was log-transformed LAR because the empirical LAR distribution was markedly right-skewed.

Our mortality rate of 30.7% in the analytic cohort is higher than mortality rates in unselected ischemic stroke populations, but it is plausible for an ICU cohort restricted to patients with both lactate and albumin measured early after ICU admission. The MIMIC-IV ischemic stroke ICU study reported a 28-day mortality rate of 34.9% among included ICU patients (9). However, we emphasize that in-hospital mortality and 28-day mortality are distinct endpoints with different follow-up windows, so direct quantitative comparisons should be avoided.

The association between LAR and mortality is biologically plausible. Lactate is a non-specific marker that may be elevated not only by tissue hypoperfusion but also by adrenergic stimulation, seizures, infection, hepatic or renal dysfunction, and certain medications (16). In ischemic stroke, lactate may also be linked to cerebral metabolic stress and the systemic consequences of severe neurological injury (17). Patients with high lactate after ICU admission may therefore represent a subgroup with more severe acute physiological derangement.

Albumin is similarly non-specific, influenced by chronic disease, systemic inflammation, fluid status, hepatic synthetic function, renal loss, nutritional status, and capillary leakage. In acute stroke, albumin has been associated with outcome and may reflect both pre-existing vulnerability and acute illness response (3, 4).

The lactate-to-albumin ratio therefore represents an empirically derived composite biomarker that integrates multiple physiological derangements, rather than a specific measure of cerebral metabolic stress or physiological reserve. This integration may explain why LAR remained associated with mortality after adjustment for severity scores. APACHE IV, GCS, and SOFA capture important aspects of physiology, organ dysfunction, and neurological status, but they may not fully capture the interaction between metabolic stress and systemic reserve (18–20).

The AUC of 0.628 deserves specific attention. In clinical prediction research, AUC values between 0.6 and 0.7 generally indicate weak or modest discrimination rather than strong predictive performance (21). The AUC indicates that LAR is better than chance but insufficient as a stand-alone classifier.

A modest AUC can still be clinically meaningful when the biomarker is inexpensive, readily available, objective, and biologically interpretable. In practice, clinicians rarely make decisions based on a single biomarker. LAR could be used as one component of early risk assessments, particularly when interpreted together with GCS, SOFA, APACHE IV, ventilatory status, renal dysfunction, hemodynamics, imaging findings, and treatment context. The most clinically relevant implication of our ROC analysis is therefore not that LAR should replace severity scores, but that elevated LAR identifies a higher-risk phenotype that deserves attention. The strong point estimate in AKI patients may reflect impaired lactate clearance and albumin loss/dilution in renal dysfunction, but the interaction was not statistically significant (*p* = 0.160). Component analyses indicated that the prognostic performance of LAR was driven primarily by lactate rather than albumin; LAR did not outperform lactate alone, suggesting that its value lies in conceptual integration rather than superior discrimination.

A central methodological choice in this study was the use of log-transformed LAR. This choice was based on observed data characteristics rather than an attempt to select a statistically favorable result. In the analytic cohort, LAR was clearly right-skewed, with a small number of very high values. When raw LAR was modeled continuously, the association with in-hospital mortality was directionally consistent but not statistically significant after full adjustment. This suggests that the raw variable may be influenced by extreme values or may not represent the relationship efficiently on the logit scale.

Log transformation of right-skewed biomarkers can reduce the leverage effect of extremely high values. A change from 0.3 to 0.6 and from 1.5 to 3.0 both represent doubling, but raw-scale modeling treats them as different absolute changes. For ratio biomarkers such as LAR, relative change may be more clinically meaningful than absolute change. The LAR doubling estimate further supports this interpretation. By reporting both log(LAR) and LAR doubling, we provide a statistically appropriate and clinically understandable representation of exposure.

Several limitations should be acknowledged. First, selection bias may have been introduced because the analytical cohort was restricted to patients with both first lactate and first albumin measured after ICU admission. Second, stroke-specific prognostic information was not available. We could not adjust for the National Institutes of Health Stroke Scale (NIHSS) score, infarct volume, collateral status, or reperfusion therapies, which are major determinants of outcome in ischemic stroke and may confound the observed association. Third, this was a retrospective observational study. Associations cannot be interpreted as causal, and residual confounding is inevitable. Although we adjusted for GCS as a measure of neurological severity, it is an imperfect proxy for stroke-specific severity measures such as the NIHSS, infarct volume, and stroke subtype. Residual confounding by unmeasured neurological severity and stroke characteristics therefore remains possible. Fourth, the analytic cohort was restricted to patients with both first lactate and first albumin measured. Of 5,414 diagnosis-eligible patients, only 759 had both measurements available. This may introduce selection bias because lactate and albumin are more likely to be measured in sicker patients or for those with suspected systemic instability. Fifth, only the first laboratory measurements were analyzed. LAR may change substantially during ICU care, and dynamic trajectories could provide additional prognostic information beyond a single baseline value. Complete-case multivariable analysis included 576 of 749 patients because APACHE IV, GCS, or SOFA scores were missing in some patients. Although the number of events remained adequate for the model, complete-case analysis can introduce bias if missingness is not random. Lactate and albumin concentrations may be influenced by ICU interventions, and the timing of measurements relative to these interventions cannot be verified. Resuscitation-induced hemodilution may lower albumin, while effective resuscitation may lower lactate, both of which could alter LAR independently of underlying disease severity. Future studies with time-stamped laboratory and treatment data are needed. Finally, binary variables such as AKI, ventilation, diabetes, and hypertension were recoded from 1/missing patterns, assuming that missing values indicated the absence of the condition or support. This assumption may lead to misclassification.

## Conclusion

In adult ICU patients with ischemic stroke, higher LAR was independently associated with increased in-hospital mortality. The association persisted after adjustment for age, sex, APACHE IV score, GCS, and SOFA score. Each two-fold increase in LAR was associated with higher mortality odds, and the highest LAR quartile was associated with more than twice the adjusted odds of in-hospital death compared with the lowest quartile. LAR showed modest discriminatory ability and should be considered an accessible adjunctive risk marker rather than a stand-alone prognostic tool. The absence of significant non-linearity and consistent results across sensitivity analyses support the use of log-transformed LAR as the main exposure representation. Prospective validation and studies incorporating stroke-specific severity, treatment information, functional outcomes, and dynamic LAR trajectories are warranted.

## Data Availability

The original contributions presented in the study are included in the article/Supplementary material, further inquiries can be directed to the corresponding author.

## Supplementary material

SUPPLEMENTARY FIGURE S1 Flow diagram of patient selection and cohort derivation.

SUPPLEMENTARY FIGURE S2 Directed acyclic graph (DAG) illustrating the conceptual causal framework for the association between admission lactate-to-albumin ratio (LAR) and in-hospital mortality.

SUPPLEMENTARY FIGURE S3 Distribution of the lactate-to-albumin ratio (LAR) in the complete-case cohort.
