# Supplementary material for: Association between lactate-to-albumin ratio and in-hospital mortality in ICU patients with acute ischemic stroke: a retrospective cohort study
Source: Front Neurol. 2026 Sep 17;17:1908031. doi: 10.3389/fneur.2026.1908031 (PMC13626895; doi:10.3389/fneur.2026.1908031)
Supplement: Supplementary file 1 [file Table_1.DOCX]

*Supplementary Table 1. Extended clinical and laboratory characteristics according to LAR quartiles*

| **Characteristic** | **Q1 N = 188** | **Q2 N = 187** | **Q3 N = 187** | **Q4 N = 187** | **P value** |
| --- | --- | --- | --- | --- | --- |
| Heart rate, beats/min | 82.0 (71.0–95.5) | 86.0 (72.0–100.0) | 87.0 (73.0–101.0) | 92.5 (75.0–109.0) | 0.002 |
| Respiratory rate, breaths/min | 18.0 (16.0–21.5) | 19.0 (16.0–22.0) | 19.0 (16.0–23.0) | 19.0 (16.0–25.0) | 0.177 |
| Serum sodium, mmol/L | 139.0 (136.0–141.0) | 139.0 (136.0–142.0) | 139.0 (136.0–143.0) | 139.0 (135.0–143.0) | 0.632 |
| Serum potassium, mmol/L | 3.9 (3.5–4.3) | 4.0 (3.6–4.3) | 3.8 (3.5–4.2) | 4.0 (3.4–4.5) | 0.128 |
| Blood urea nitrogen, mg/dL | 18.0 (11.5–34.0) | 19.0 (13.0–27.0) | 20.5 (13.0–34.0) | 25.5 (16.0–37.0) | <0.001 |
| Serum creatinine, mg/dL | 1.01 (0.75–1.68) | 0.94 (0.78–1.36) | 1.00 (0.73–1.64) | 1.31 (0.84–2.10) | <0.001 |
| White blood cell count, K/mcL | 11.1 (8.4–14.3) | 11.8 (8.3–15.5) | 12.3 (9.2–16.8) | 14.5 (10.1–19.4) | <0.001 |
| Hemoglobin, g/dL | 11.7 (9.9–13.3) | 12.0 (9.7–13.6) | 12.0 (10.1–13.8) | 11.5 (9.6–13.1) | 0.224 |
| Platelet count, K/mcL | 202.5 (157.0–249.0) | 188.0 (143.0–248.0) | 195.0 (137.0–253.0) | 179.0 (112.0–251.0) | 0.064 |
| Diabetes mellitus, n (%) | 13 (6.9%) | 15 (8.0%) | 12 (6.4%) | 24 (13%) | 0.103 |
| Hypertension, n (%) | 80 (43%) | 58 (31%) | 58 (31%) | 60 (32%) | 0.048 |
| ICU length of stay, days | 5.7 (2.8–11.6) | 5.8 (2.9–11.1) | 4.4 (2.6–9.7) | 6.4 (3.1–11.1) | 0.139 |
| Hospital length of stay, days | 10.9 (6.1–19.0) | 11.0 (5.9–19.2) | 11.1 (5.0–18.8) | 10.9 (5.3–17.0) | 0.830 |
| Abbreviations: LAR, lactate-to-albumin ratio; ICU, intensive care unit. | | | | | |
