# Supplementary material for: Association between lactate-to-albumin ratio and in-hospital mortality in ICU patients with acute ischemic stroke: a retrospective cohort study
Source: Front Neurol. 2026 Sep 17;17:1908031. doi: 10.3389/fneur.2026.1908031 (PMC13626895; doi:10.3389/fneur.2026.1908031)
Supplement: Supplementary file 2 [file Table_2.DOCX]

*Supplementary Table 2. Sensitivity analyses of the association between LAR and in-hospital mortality*

| **Analysis** | **Effect estimate** | **P value** | **Interpretation** |
| --- | --- | --- | --- |
| log(LAR), continuous | 1.44 (1.09–1.91) | 0.010 | Primary exposure definition |
| LAR doubling | 1.29 (1.06–1.56) | 0.010 | Effect estimate for each two-fold increase in LAR |
| Raw LAR, continuous | 1.23 (0.97–1.56) | 0.083 | Supplementary analysis using untransformed LAR |
| LAR Q4 vs Q1 | 2.16 (1.20–3.89) | 0.010 | Highest versus lowest LAR quartile |
| RCS overall association | — | 0.041 | Overall association between log(LAR) and in-hospital mortality |
| RCS nonlinearity | — | 0.463 | No statistically significant evidence of nonlinearity |
| Abbreviations: LAR, lactate-to-albumin ratio; OR, odds ratio; CI, confidence interval; RCS, restricted cubic spline; APACHE IV, Acute Physiology and Chronic Health Evaluation IV; SOFA, Sequential Organ Failure Assessment. | | | |
