# Supplementary material for: Association between lactate-to-albumin ratio and in-hospital mortality in ICU patients with acute ischemic stroke: a retrospective cohort study
Source: Front Neurol. 2026 Sep 17;17:1908031. doi: 10.3389/fneur.2026.1908031 (PMC13626895; doi:10.3389/fneur.2026.1908031)
Supplement: Supplementary file 3 [file Table_3.DOCX]

**Supplementary Table S3 ICD-10 diagnostic codes used to identify adult ICU patients with ischemic stroke**

| ICD-10-CM | Description |
| --- | --- |
| I63.0 | Cerebral infarction due to thrombosis of precerebral arteries |
| I63.1 | Cerebral infarction due to embolism of precerebral arteries |
| I63.2 | Cerebral infarction due to unspecified occlusion/stenosis of precerebral arteries |
| I63.3 | Cerebral infarction due to thrombosis of cerebral arteries |
| I63.4 | Cerebral infarction due to embolism of cerebral arteries |
| I63.5 | Cerebral infarction due to unspecified occlusion/stenosis of cerebral arteries |
| I63.8 | Other cerebral infarction |
| I63.9 | Cerebral infarction, unspecified |
